# Investigating the moderators and mediators of an effective sleep intervention in the Prevention of Overweight in Infancy (POI) randomized controlled trial: Exploratory analyses

**DOI:** 10.1111/cob.12516

**Published:** 2022-03-16

**Authors:** Louise Fangupo, Jillian Haszard, Barbara Galland, Barry Taylor, Anne‐Louise Heath, Dione Healey, Kim Meredith‐Jones, Rachel Sayers, Burt Hatch, Rachael Taylor

**Affiliations:** ^1^ Department of Medicine University of Otago Dunedin New Zealand; ^2^ Biostatistics Centre University of Otago Dunedin New Zealand; ^3^ Department of Women and Children's Health University of Otago Dunedin New Zealand; ^4^ Department of Human Nutrition University of Otago Dunedin New Zealand; ^5^ Department of Psychology University of Otago Dunedin New Zealand; ^6^ School of Nursing, Otago Polytechnic Dunedin New Zealand; ^7^ ISN Innovations, Institute for Social Neuroscience Melbourne Victoria Australia

**Keywords:** intervention, obesity, paediatrics, sleep

## Abstract

The Prevention of Overweight in Infancy (POI) sleep intervention halved obesity risk at 2 years of age. However, the intervention mechanisms are unclear. Consequently, the objective of the current work was to use exploratory analyses to investigate potential moderators and mediators of the sleep intervention on obesity outcomes at age 2 years. Data were collected between 2009 and 2012. The effect of demographic and study design variables on body mass index *z*‐score (BMI *z*‐score) and obesity was compared in moderator subgroups at 2 years of age (*n* = 683, 85%). Mediating effects of child and parent–household variables assessed whether the sleep intervention resulted in meaningful changes in the mediating variable (defined as changes which were statistically significant [*p* < .05] or where the effect size was ≥0.15 SD), followed by assessing relationships with obesity outcomes. The sleep intervention appeared most effective in children in higher deprivation areas (effect on BMI *z*‐score −0.25 [−0.53, 0.04], effect on obesity odds ratio [OR] 0.43 [0.16, 1.13]), and with mothers of non‐European, non‐Māori ethnicity (effect on BMI *z*‐score −0.27 [−0.73, 0.20], effect on obesity OR 0.13 [95% confidence interval 0.01, 1.11]). This suggested moderation by deprivation and ethnicity. Aspects of sleep improved meaningfully in children after intervention but did not significantly relate to obesity outcomes, and other outcomes were not meaningfully affected by the sleep intervention. Thus, mediation was not indicated. Overall, the POI sleep intervention improved obesity outcomes at 2 years, and the current work identified some potential moderators, but no mediators.

**What is already known about this subject?**

- Observational studies have found that short sleep duration and poor sleep quality are associated with an increased risk of childhood obesity.
- The Prevention of Overweight in Infancy (POI) sleep intervention which was conducted antenatally and in early infancy, halved obesity risk at 2 years of age. However, the mechanisms behind this effect are unclear.
- Moderation and mediation analyses are useful for exploring potential intervention mechanisms.

**What does this study adds?**

- Exploratory analyses, investigating a range of potential moderators and mediators of the Prevention of Overweight in Infancy (POI) sleep intervention on body mass index *z*‐score and obesity at 2 years of age, were undertaken.
- Some potential moderators were identified, including maternal ethnicity and area‐level deprivation.
- Aspects of sleep improved in children following the POI sleep intervention. However, they did not significantly relate to obesity outcomes. Other outcomes were not meaningfully impacted by the sleep intervention. Thus, no mediators were identified.

## 1 INTRODUCTION

Internationally, approximately 38 million children under 5 years of age are affected by overweight or obesity.^1^ Excess weight in childhood adversely affects physical and psychological health, contributes to behavioural and emotional difficulties, reduces educational attainment,^2^ and often persists into adulthood,^3^ where it is associated with an increased risk of serious non‐communicable diseases.^4^ In recent years a number of interventions have targeted obesity prevention from the earliest days of life,^5^ when metabolic and behavioural patterns are still developing. They have had only relatively modest effects,^5^, ^6^ which has led to calls for testing alternative approaches to the more commonly targeted lifestyle behaviours of diet, physical activity and media use.^6^, ^7^

Sleep is an alternative approach which is supported by consistent observational evidence indicating that short sleep duration^8^ and poor sleep quality^9^ are associated with an increased risk of childhood obesity, although interventions to date are limited.^10^ Our Prevention of Overweight in Infancy (POI) trial^11^ showed that a brief sleep intervention in infancy substantially reduced the risk of obesity at 2^12^ years of age. However, earlier analyses did not specifically report differences in lifestyle behaviours of interest (such as diet,^13^, ^14^ sleep,^12^, ^14^, ^15^ and activity^12^, ^14^, ^16^) for the groups that received the sleep intervention compared to the groups that did not. Thus, there is a need for new analyses, which explore the potential mechanisms behind the observed benefits of the POI sleep intervention on obesity.

Moderation and mediation analyses (Figures S1 and S2) provide a method for identifying potential causal pathways with moderation describing *when* or *for whom* an independent variable most strongly causes a dependent variable, and mediation explaining *how* and *why* the independent variable causes the effect on the dependent variable.^17^ In this context, there were several factors that we sought to explore through moderation and mediation.

With regard to moderation (Figure S1), we were aware that mothers who participated in the POI study were predominantly well‐educated and of New Zealand European ethnicity, and that fewer families lived in areas of high deprivation than is observed in the New Zealand population as a whole.^12^, ^14^ It is possible that the intervention was received differently by, or had different impacts on, participating families with other demographic characteristics. Furthermore, outcomes could be moderated by certain study design features. For example, different levels of support were available to families within the sleep intervention, and the POI study was designed as a 2 × 2 factorial trial, meaning that some participants in the sleep group also received a Food, Activity and Breastfeeding (FAB) intervention.

With regard to mediation (Figure S2), we hypothesized that there were a range of ‘child’ and ‘parent–household’ factors with potential to mediate the effects of the POI sleep intervention on obesity outcomes. The sleep intervention educated parents about normal infant sleep development, and emphasized that parents should give infants opportunities to learn to settle to sleep unaided^11^ in an effort to promote the ability for children to self‐regulate their sleep behaviours. As such, we hypothesized that this focus may have influenced ‘child factors’ such as how they slept, and their general self‐regulatory abilities, as well as a range of ‘parent–household factors’ potentially associated with childhood obesity, such as parenting style,^18^ stress related to parenting,^19^ parental feeding practices,^20^ and parental mental health.^21^

The objectives of the current work were to determine whether demographic and study design factors moderated, and child and parent–household factors mediated, the effect of the POI sleep intervention on obesity outcomes at 2 years of age. All analyses are of an exploratory nature, for the purpose of setting directions for future early childhood sleep interventions.

## 2 MATERIALS AND METHODS

### 2.1 POI trial

Information about the POI trial and the subsequent follow‐up study are available elsewhere.^11^, ^22^ In brief, POI was a 4‐arm randomized controlled trial (RCT) designed to test the effectiveness of interventions designed to reduce the risk of obesity in early life. The four study arms were Usual Care (control); Sleep; FAB; and Combination (Sleep and FAB combined). All pregnant women booking into the only birthing unit in Dunedin, New Zealand, from May 2009 to December 2010 were eligible if they were aged ≥16 years, <34 weeks' gestation, able to communicate in English or Te Reo Māori (indigenous language of New Zealand) and planned to live locally for 2 years. After assessment, 1458 women were eligible; of these, 611 (42%) declined to participate. Infants were excluded after birth if gestation was <36.5 weeks, or if they had a congenital abnormality or physical–intellectual disability likely to affect feeding, physical activity or growth. The final sample size was 802. Retention was 86% at 2 years. Families were randomly allocated to 1 of the four study arms within 6 strata depending on area‐level deprivation^23^ (3 levels) and parity (2 levels) by using a block size of 12. Participants in all four arms received standard government‐funded well child care.^24^ Those in the Usual Care group did not receive any additional intervention. Families in the intervention groups received additional guidance and support related to Sleep, FAB or both. Those delivering and receiving interventions could not be blinded, but the main outcome measurements were performed by researchers blinded to group allocation. Ethical approval for the POI trial was obtained from the Lower South Regional Ethics Committee (LRS/08/12/063). Written informed consent was obtained from the parent–guardian of all child participants. The trial was registered at clinicaltrials.gov as NCT00892983.

### 2.2 POI sleep intervention

Families who received the POI sleep intervention (those in the Sleep and Combination groups) received antenatal information delivered by a single group session (up to 1 h long, typically during the third trimester), followed by one home visit (30–60 min) at 3 weeks postpartum with a researcher with infant sleep training. The messages delivered on each of these occasions have been summarized elsewhere.^15^ In summary, the antenatal session educated all mothers and some partners (mostly fathers) about normal developmental patterns of infant sleep and emphasized settling to sleep unaided.^15^ Safe sleep practices to prevent Sudden Unexpected Death in Infancy were also promoted. Parents were given a ‘Tip Sheet for Developing Healthy Sleep Patterns’ to take home. The individual sessions at the 3‐week home visit reinforced the antenatal sleep education and were conducted with the aid of a 15‐page booklet covering the key messages. Parents were provided with a copy of the booklet and encouraged to refer back to it as often as necessary. At 6 months, and 1 and 1.5 years, parents who indicated that their child's sleep was problematic were offered additional assistance from a research nurse who worked alongside the family to implement one or more of four approaches, which differed in complexity. At the first level, families received simple advice (e.g., about changes to sleeping arrangements or feeding). The second level involved advice on settling techniques only. The third level involved a partial sleep intervention, and the fourth level a full sleep intervention.^11^ Each family chose the level, or combination of levels, which they felt were most appropriate for their family, and used the relevant tools as often as they liked.

### 2.3 Measurements

Comprehensive details of all measurement procedures are described in Table 1. To summarize briefly here, anthropometric measures were obtained by trained measurers blinded to intervention status at 2 years of age. Family demographic characteristics were collected by questionnaire at baseline (late pregnancy). Children's sleep and other behaviours were assessed by actigraphy, questionnaires or laboratory assessments at time points between the ages of 1 and 2 years.^11^, ^22^ Similarly, parent and household factors were assessed by questionnaire between the ages of 1 and 2 years, as described in Table 1.

**TABLE 1:** Methods of measurement for potential moderating and mediating factors in the Prevention of Overweight in Infancy (POI) study

Factor	Variable	Method of data collection and timing	Measurement procedures and use of data
Obesity outcomes			
Anthropometry	Child's weight Child's length	Trained measurers, 2 years Trained measurers, 2 years	To determine child's body mass index (BMI) *z*‐score according to WHO growth standards,^39^ duplicate measures of weight (Tanita WB‐100 MA/WB‐110 MA) and length (Harpenden stadiometer, Holtain Ltd) were obtained by trained measurers following World Health Organization (WHO) protocols.^40^ Obesity was defined as BMI ≥95th percentile
Potential moderating factors			
Demographic characteristics	Infant sex Household address Parity Maternal education level Maternal ethnicity Maternal height, weight	Hospital records, post‐natally Questionnaire, baseline^a^ Questionnaire, baseline^a^ Questionnaire, baseline^a^ Questionnaire, baseline^a^ Questionnaire, baseline^a^	To determine male or female infant sex To determine New Zealand Deprivation Index 13 (23) score. Scores range from 1 (least deprived) to 10 (most deprived) and reflect the extent of material and social deprivation in the geographical area in which each family live To determine maternal primi‐ or multi‐parity To describe whether mothers had a tertiary qualification, or not To describe maternal ethnicity as European, Māori or Other To calculate maternal self‐reported, pre‐pregnancy BMI in kg/m^2^ and determine pre‐pregnancy obesity (BMI ≥30 kg/m^2^)
Study design	Intervention status Additional support in sleep intervention	Study records, post‐intervention Study records, post‐intervention	To determine whether participants received the Food, Activity and Breastfeeding (FAB) intervention To determine whether those in the sleep intervention received any of the additional support, which was available if required
Potential mediating factors: Child			
Sleep characteristics	Sleep onset Sleep offset Nocturnal total sleep time Time spent awake after sleep onset Overnight sleep efficiency Number of night awakenings Infant sleep problem score	Actigraphy, 1 and 2 years Actigraphy, 1 and 2 years Actigraphy, 1 and 2 years Actigraphy, 1 and 2 years Actigraphy, 1 and 2 years Actigraphy, 1 and 2 years Questionnaire, 1.5 and 2 years	Actigraphy outcomes were collected using Actical (Mini‐Mitter, Bend, OR) accelerometers, which children wore on elastic belts around their waist for 24 h per day for 7 days. Data were cleaned and scored using an automated script developed in MATLAB (Mathworks, Natick, MA)^41^ This count‐scaled algorithm estimated sleep onset (start of the first 15 continuous minutes of sleep preceded by 5 min of awake) and offset (last of 15 continuous minutes of sleep followed by 5 min of awake) specific to each individual for each day Calculated as the duration of time overnight between sleep onset and offset, excluding time awake^42^ Defined as the sum (in min) of all overnight awakenings. An awakening is defined as a period of time after sleep onset and before sleep offset where there are 5 continuous minutes of wake epochs preceded and followed by 15 min of sleep epochs Defined as the ratio of nocturnal total sleep time to sleep period time (i.e., time between sleep onset and offset including time awake) × 100. Defined as the total number of night awakenings between sleep onset and offset Parents rated whether their child's sleep was a problem on an eight‐point scale from 0 (no problem) to 7 (large problem)
Temperament		Questionnaire, 2 years	Assessed using 6 subscales from the Colorado Childhood Temperament Inventory (CCTI): sociability, emotionality, activity, attention span persistence, reaction to food and soothability.^43^ Each subscale contained five items, which parents scored using a five‐point scale from 1 (not at all like my child) to 5 (a lot like my child). Higher scores indicate higher levels of each behaviour
Potential mediating factors—parent/household			
Maternal mental health	Depression Depression, anxiety and stress	Questionnaire, 1 year Questionnaire, 2 years	The Edinburgh Postnatal Depression Scale (EDPS)^44^ was used to measure postnatal depression. The EDPS is a 10‐item scale, which asks mothers how they have felt in the past 7 days. Mothers scored each item on a four‐point scale from 0 (least indicative of a postnatal depression symptom) to 3 (most indicative of a postnatal depression symptom). High total scores indicated that mothers were likely to be suffering from depressive illness Measured using the 21‐item Depression Anxiety Stress Scales (DASS‐21).^45^ Items in DASS were scored on a four‐point scale from 0 (did not apply to me) to 3 (applied to me very much or most of the time). Higher overall scores indicated greater depression, anxiety and stress
Parenting	Parenting style	Questionnaire, 2 years	Baumrind's concept,^46^ which has three constructs: authoritarian, permissive and authoritative, was used to measure parenting style. Higher scores indicate higher usage of each parenting style
	Parental feeding practices	Questionnaire, 1.5 years Questionnaire, 2y	Seven subscales from the Comprehensive Feeding Practices Questionnaire (CFPQ)^47^ (Emotion Regulation, Food as Reward, Pressure to Eat, Restriction for Health, Modelling, Monitoring and Healthy Environment) were used to measure parental feeding practices. Each subscale consisted of 3–4 items, each of which were measured using a five‐point scale (either ‘never, rarely, sometimes, mostly, always’ or ‘disagree, slightly disagree, neutral, slightly agree, agree’). Higher scores indicated greater use of the relevant parental feeding practice Six factors from Murashima's feeding control instrument^48^ were used to assess parental feeding practices (high control, high contingency, child‐centred feeding, encourage nutrient‐dense foods, discourage energy‐dense foods, mealtime behaviour). Each factor consisted of several items, and each item was scored using a five‐point scale from 1 (never) to 5 (always). Higher scores indicated greater use of the relevant parental feeding practice.
	Discipline practices	Questionnaire, 1.5 years	Positive discipline practices were assessed using an age‐appropriate list of behaviours developed by the POI research team.^49^ Parents were asked to indicate how often over the past 7 days they had employed each behaviour to get their child to do, or stop doing, something. Higher scores indicated greater use of positive discipline practices
	Family quality of life	Questionnaire, 1 year	Family quality of life was measured using four subscales from the Beach Centre Family Quality of Life Scale (family interaction, parenting, emotional wellbeing and physical wellbeing^50^). Items in the subscales were answered using a five‐point scale from 1 (very dissatisfied) to 5 (very satisfied). Higher scores indicated greater family quality of life

^a^ The baseline questionnaire was completed by the mother in late pregnancy.

### 2.4 Statistical analysis

Stata 16.1 (StataCorp, TX) was used for all analyses. No adjustments for multiple comparisons were made. Residuals of all linear regression models were plotted and visually assessed for heteroscedasticity and normality.

Moderation effects for demographic and study variables (sex, deprivation, parity, maternal education, maternal ethnicity, maternal obesity, FAB intervention and additional sleep support) were assessed by subgroup analyses. These were exploratory analyses (not pre‐specified) and while tests of interaction were undertaken, the study is likely to be underpowered to detect statistically significant (*p* < .05) interactions.^25^ Linear regression models were used to estimate the effect of the sleep intervention by subgroup, with body mass index (BMI) *z*‐score as the outcome variable and intervention group as the predictor variable. An interaction term between intervention group and demographic subgroup was then included in the model to determine the *p*‐value for interaction. For each subgroup, the mean difference in BMI *z*‐score for those who received the sleep intervention compared to those who did not receive a sleep intervention was calculated along with the 95% confidence interval (CI). Logistic regression models were used in the same way for obesity outcomes with odds ratios (OR) reported.

All potential mediating variables (except for sleep variables) were standardized to be in units of SD to allow identification of the variables that were most strongly affected by the sleep intervention. Potential mediating variables were decided a priori to be: sleep variables (total sleep time, time awake after sleep onset, sleep efficiency, number of night wakings and sleep problem score), child temperament at 2 years of age, maternal depression, parental stress, parenting style, parental feeding practices, discipline strategies and family quality of life. Mediation was explored using a three‐step process^26^: (1) First, we determined whether the potential mediating variable differed by sleep intervention group. Linear regression models were run with the potential mediating variable as the outcome and intervention group as the predictor. Mean differences, 95% CI and *p*‐values were calculated with adjustment for the study design factors of parity, deprivation and whether they had received the FAB intervention; (2) If a potential mediating variable differed in a meaningful way by intervention group, then the relationship with BMI *z*‐score was determined. ‘Meaningful’ here was decided a priori to be if the relationship was statistically significant (*p* < .05), or if the effect size was at least 0.15 SD.^27^ Linear regression models were used with BMI *z*‐score as the outcome variable, the potential mediating variable as the predictor variable and adjustment for sleep intervention, FAB intervention, parity and deprivation; and if the sleep intervention had a meaningful effect on a potential mediating variable, and was related to BMI *z*‐score in a meaningful way (decided a priori to be statistically significant or a mean difference of 0.05 or greater), then this variable was considered a mediator. The extent of mediation was assessed by the percent of the effect size that was reduced after adjustment for the mediator. The same method was used for obesity outcomes but using logistic regression and reporting odds ratios.

## 3 RESULTS

Overall, the prevalence of overweight and obesity among children in the POI study at 2 years of age was 40.0% (273/683) while the prevalence of obesity alone was 16.5% (114/683).^12^

### 3.1 Moderation analyses

The results of the moderation analysis for BMI *z*‐score and obesity are displayed in Table 2. Overall, the sleep intervention improved BMI *z*‐score (mean difference, 95% CI: −0.15, −0.28 to −0.01) and obesity (OR, 95% CI: 0.52, 0.34–0.80) at 2 years of age.

**TABLE 2:** Demographic and study design factors and assessment of effect moderation on obesity outcomes at 2 years of age

	*n* (%) at 2 years	Mean difference (95% confidence interval [CI]) body mass index (BMI) *z*‐score at 2 years for those who received the sleep intervention compared to those who did not	*p*‐value for interaction^a^	Odds ratio (95% CI) for obesity at 2 years in those who received the sleep intervention compared to those who did not	*p*‐value for interaction^a^
Overall	683	−0.15 (−0.28, −0.01)		0.52 (0.34, 0.80)	
Sex			.728		.416
Male	351 (51.4)	−0.13 (−0.32, 0.06)		0.44 (0.25, 0.79)	
Female	332 (48.6)	−0.18 (−0.37, 0.02)		0.63 (0.34, 1.16)	
Deprivation^b^			.707		.889
Low	250 (37.0)	−0.09 (−0.31, 0.14)		0.57 (0.28, 1.15)	
Medium	289 (42.8)	−0.14 (−0.36, 0.07)		0.55 (0.30, 1.03)	
High	137 (20.3)	−0.25 (−0.53, 0.04)		0.43 (0.16, 1.13)	
Parity			.415		.525
Primiparous	322 (47.1)	−0.20 (−0.41, 0.01)		0.45 (0.24, 0.84)	
Multiparous	361 (52.9)	−0.09 (−0.27, 0.09)		0.60 (0.34, 1.05)	
Maternal education^c^			.951		.861
No tertiary qualification	239 (35.2)	−0.13 (−0.36, 0.10)		0.51 (0.26, 1.00)	
Tertiary qualification	440 (64.8)	−0.14 (−0.30, 0.03)		0.55 (0.32, 0.94)	
Maternal ethnicity			.780		‐^d^
European	590 (86.4)	−0.14 (−0.29, 0.00)		0.56 (0.36, 0.88)	
Māori	32 (4.7)	0.01 (−0.59, 0.60)		0.75 (0.09, 6.11)	
Other	61 (8.9)	−0.27 (−0.73, 0.20)		0.13 (0.01, 1.11)	
Maternal obesity pre‐pregnancy			.671		.977
No obesity	591 (86.9)	−0.12 (−0.26, 0.03)		0.53 (0.34, 0.85)	
Obesity	89 (13.1)	−0.21 (−0.57, 0.16)		0.53 (0.19, 1.44)	
Intervention			.512		.743
No FAB	340 (49.8)	−0.10 (−0.29, 0.09)		0.56 (0.31, 1.03)	
FAB	343 (50.2)	−0.19 (−0.39, 0.00)		0.49 (0.27, 0.87)	
Additional support in sleep intervention^e^			.369		.723
No additional support	230 (69.9)	−0.18 (−0.33, −0.03)		0.50 (0.31, 0.81)	
Additional support	99 (30.1)	−0.08 (−0.28, 0.12)		0.57 (0.30, 1.08)	

Abbreviation: FAB, food, activity and breastfeeding (intervention group).

^a^ *p*‐value for interaction is presented but caution is advised as adjustment for multiple tests was not undertaken and there may not be power to detect interactions. We recommend comparing the effect sizes presented.

^b^ Seven participants were missing deprivation data.

^c^ Four participants were missing maternal education data.

^d^ Not estimable. All mothers of Māori ethnicity had child without obesity at 2 years of age.

^e^ Additional support in the sleep intervention was analysed by splitting the sample into three categories: (1) not randomized to sleep intervention; (2) randomized to basic sleep intervention and (3) randomized to basic sleep intervention plus additional support around sleep. Category (1) is the reference category for estimates of mean differences in BMI *z*‐score and odds ratios for obesity. Percentages here represent the percent of the sleep group that did not and did access additional support.

With regard to BMI *z*‐score, the sleep intervention appeared to be more effective in children with mothers of ‘Other’ ethnicities (mean difference −0.27, 95% CI −0.73 to 0.20), compared to in children with mothers of European (mean difference −0.14, 95% CI −0.29 to 0.00), and particularly Māori (mean difference 0.01, 95% CI −0.59 to 0.60), ethnicity although the interaction term was not significant. A similar pattern was observed for area‐level deprivation, whereby the interaction term was not significant but effect sizes suggested that the intervention was more effective among children living in areas of high deprivation (mean difference −0.25, 95% CI −0.53 to 0.04) than in those living in areas of medium (mean difference −0.14, 95% CI −0.36 to 0.07) or low (mean difference −0.09, 95% CI −0.31 to 0.14) deprivation. The intervention also appeared to be more effective in children whose mothers were classified as obese pre‐pregnancy (mean difference −0.21 (−0.57 to 0.16) than in children of mothers who were not (mean difference −0.12, 95% CI −0.26 to 0.03).

For obesity, similar trends were observed to those described above for BMI *z*‐score. Although the interaction term was not significant, the intervention appeared to be more effective at reducing the risk of obesity among children living in areas of high (OR 0.43, 95% CI 0.16–1.13) rather than medium (OR 0.55, 95% CI 0.30–1.03) or low (OR 0.57, 95% CI 0.28–1.15) deprivation. Furthermore, and although again the interaction term was not significant, it appeared to be more effective among children of non‐Māori, non‐European mothers (OR 0.13, 95% CI 0.01–1.11) than among children with Māori (OR 0.75, 95% CI 0.09–6.11) or European (OR 0.56, 95% CI 0.36–0.88) mothers. However, unlike for BMI *z*‐score, it did not appear that the intervention was more effective for obesity among children whose mothers had pre‐pregnancy obesity (OR 0.53, 95% CI 0.19–1.44).

### 3.2 Mediation analysis: Child factors

Table 3 shows the mean differences in a variety of sleep measures as well as child temperament indicators for children who had received the POI sleep intervention compared to those who had not. Although overall differences were small, the sleep intervention appeared to have an impact on three nocturnal sleep variable outcomes measured by actigraphy at 1 year of age: decreasing time spent awake after sleep onset, increasing overnight sleep efficiency and decreasing the number of night wakings. It also reduced infant sleep problem scores at 1.5 years of age. However, the POI sleep intervention did not appear to have a meaningful impact on any aspect of child temperament at 2 years of age.

**TABLE 3:** Differences in child factors among participants who received the sleep intervention compared to those who did not

Sleep outcomes	Mean (SD)	*n*	Mean difference (95% confidence interval [CI])^a^ for those who received the sleep intervention compared to those who did not	*p*‐value^b^
Nocturnal total sleep time^c^, min				
1 year	623 (59)	396	−1.5 (−13.2, 10.2)	.799
2 years	622 (53)	297	−5.1 (−17.5, 7.2)	.413
Time spent awake after sleep onset^c^, min				
1 year	23 (7, 45)	396	−8.0 (−15.0, −1.0)	.025
2 years	13 (3, 30)	297	2.6 (−3.4, 8.6)	.393
Overnight sleep efficiency^c^, %				
1 year	95.5 (4.4)	396	1.2 (0.4, 2.1)	.004
2 years	96.8 (3.6)	297	0.0 (−0.8, 0.8)	.964
Number of night wakings^c^, *n*				
1 year	0.85 (0.69)	396	−0.14 (−0.28, −0.01)	.042
2 years	0.60 (0.57)	297	−0.02 (−0.16, 0.11)	.739
Infant sleep problem score^d^				
1.5 years	2.4 (1.7)	602	−0.3 (−0.5, 0.0)	.062
2 years	2.2 (1.5)	503	−0.2 (−0.4, 0.1)	.269

Questionnaire outcomes	Mean (SD)	*n*	Standardized^a^ mean difference (95% confidence interval [CI]) for those who received the sleep intervention compared to those who did not	*p*‐value^b^
Child temperament at 2 years				
Sociability	18.2 (4.3)	482	0.02 (−0.16, 0.21)	.789
Emotionality	10.8 (4.2)	482	0.02 (−0.16, 0.20)	.814
Activity	20.7 (3.6)	483	0.14 (−0.04, 0.32)	.121
Attention span persistence	16.1 (3.7)	483	−0.15 (−0.33, 0.03)	.105
Reaction to food	11.7 (4.7)	483	−0.02 (−0.20, 0.16)	.835
Soothability	17.6 (3.4)	483	−0.13 (−0.30, 0.05)	.170

^a^ Adjusted for parity, deprivation and whether they received the FAB intervention. Standardized mean differences are presented for all questionnaire measures but not sleep outcomes.

^b^ *p*‐values are not adjusted for multiple tests. We recommend using the standardized mean differences and precision of these estimates (95% CI) to judge whether an association is meaningful.

^c^ From actigraphy. Time spent awake after sleep onset presented as median (25th, 75th percentile), with differences in the median estimated using quantile regression.

^d^ From questionnaire (eight‐point scale from 0 = no problem to 7 = large problem).

### 3.3 Mediation analysis: Parent and household factors

Table 4 demonstrates the standardized mean difference in parent and household factors for participants who received the sleep intervention compared to those who did not. The sleep intervention did not appear to have any impact on parental feeding practices (measured at 1.5 and 2 years), parenting style (2 years) or parental stress (2 years). However, at 1 year, the sleep intervention reduced maternal depression (standardized mean difference −0.16, 95% CI −0.32 to −0.01), although this effect was no longer apparent at 2 years (standardized mean difference −0.04, 95% CI −0.22, 0.14). Those who had received the sleep intervention used more positive discipline strategies at 1.5 years (standardized mean difference 0.21, 95% CI 0.05–0.37). Finally, the sleep intervention improved family interaction at 1 year (standardized mean difference 0.15, 95% CI 0.00–0.31). Evidence of improvements in other family quality of life indicators was not observed.

**TABLE 4:** Differences in parent and household factors for those who received the sleep intervention compared to those who did not

	Mean (SD)^a^	*n*	Standardized^b^ mean difference (95% confidence interval [CI]) for those who received the sleep intervention and those who did not	*p*‐value^c^
Maternal depression				
1 year (EDPS)	1.5 (0.4)	652	−0.16 (−0.32, −0.01)	.038
2 years (DASS)	3.2 (4.7)	501	−0.04 (−0.22, 0.14)	.656
Parental stress at 2 years				
Anxiety (DASS)	2.2 (3.2)	501	−0.12 (−0.30, 0.05)	.175
Stress (DASS)	8.3 (6.3)	501	−0.03 (−0.21, 0.15)	.737
Parenting style at 2 years				
Authoritative	4.3 (0.4)	498	0.06 (−0.12, 0.24)	.494
Authoritarian	1.7 (0.4)	498	0.04 (−0.14, 0.21)	.672
Permissive	2.1 (0.6)	498	0.04 (−0.13, 0.22)	.617
Parental feeding practices at 1.5 years				
Emotion regulation	2.0 (0.7)	606	0.00 (−0.16, 0.16)	.983
Food as a reward	1.7 (0.9)	606	0.08 (−0.08, 0.24)	.339
Pressure to eat	2.5 (0.9)	604	0.05 (−0.11, 0.21)	.512
Restriction for health	3.2 (1.1)	601	−0.09 (−0.25, 0.08)	.301
Modelling	4.2 (0.8)	605	−0.11 (−0.27, 0.05)	.192
Monitoring	4.5 (0.7)	604	0.13 (−0.03, 0.29)	.106
Healthy environment	3.8 (0.4)	604	0.07 (−0.10, 0.23)	.429
Parental feeding practices at 2 years				
High control	1.7 (0.7)	487	0.07 (−0.11, 0.24)	.465
High contingency	1.5 (0.6)	487	0.09 (−0.09, 0.27)	.335
Child‐centred feeding	3.2 (0.7)	486	−0.02 (−0.19, 0.16)	.861
Encourage nutrient‐dense foods	3.4 (0.8)	485	0.02 (−0.16, 0.20)	.834
Discourage energy‐dense foods	4.2 (0.6)	486	0.03 (−0.15, 0.21)	.745
Mealtime behaviour	4.3 (0.7)	486	0.03 (−0.15, 0.21)	.740
Positive discipline strategies at 1.5 years	5.8 (1.3)	601	0.21 (0.05, 0.37)	.011
Family quality of life at 1 year				
Family interaction	4.4 (0.6)	645	0.15 (0.00, 0.31)	.058
Parenting	4.4 (0.5)	643	−0.01 (−0.17, 0.14)	.871
Emotional wellbeing	3.9 (0.9)	642	0.08 (−0.08, 0.23)	.339
Physical wellbeing	4.6 (0.5)	644	0.12 (−0.03, 0.27)	.128

Abbreviations: DASS, Depression Anxiety Stress Scale; EDPS, Edinburgh Postnatal Depression Score.

^a^ Unless otherwise stated.

^b^ Adjusted for parity, deprivation and whether they received the FAB intervention. Standardized mean differences allow us to determine which factors are most strongly affected by the intervention.

^c^ *p*‐values are not adjusted for multiple tests. We recommend using the standardized mean differences and precision of these estimates (95% CI) to judge whether an association is meaningful.

### 3.4 Potential mediators and their relationships with obesity outcomes

Table 5 displays the relationships between those factors indicated as potentially important mediators in Tables 3 and 4 and the mean difference in BMI *z*‐score and odds of obesity for every 1 SD increase in the relevant factor. The effect sizes were uniformly small, ranging from −0.05 to 0.04 for BMI *z*‐score and 0.77 to 1.12 for obesity.

**TABLE 5:** Relationships between potential mediators and body mass index (BMI) *z*‐scores at 2 years of age

Standardized predictors^a^	*n*	Mean difference (95% confidence interval [CI]) in BMI *z*‐score for each SD higher	Odds ratio (95% CI) for obesity for each SD higher
Time spent awake after sleep onset at 1 year	382	0.03 (−0.06, 0.13)	1.00 (0.76, 1.32)
Sleep efficiency at 1 year	382	−0.04 (−0.13, 0.06)	1.00 (0.76, 1.33)
Number of night wakings at 1 year	382	0.04 (−0.05, 0.13)	0.95 (0.72, 1.26)
Infant sleep problem at 1.5 years	575	−0.01 (−0.09, 0.06)	0.88 (0.70, 1.10)
Maternal depression at 1 year	602	0.04 (−0.03, 0.11)	1.12 (0.91, 1.38)
Positive discipline strategies at 1.5 years	574	−0.01 (−0.09, 0.06)	0.93 (0.75, 1.15)
Family interaction at 1 year	603	−0.05 (−0.12, 0.02)	0.77 (0.57, 1.05)

^a^ All potential mediators are standardized so are in units of SD.

Table 6 displays the results of the mediation of the sleep intervention on BMI *z*‐score and odds of obesity at 2 years. None of the potentially important mediators were found to be meaningful, with each explaining less than 6% of the effect of the sleep intervention on BMI *z*‐score mean difference and obesity OR at 2 years.

**TABLE 6:** Mediation of sleep intervention on body mass index (BMI) *z*‐score and obesity at 2 years

	*n*	Effect of sleep intervention (‘total effect’)	Effect of sleep intervention after adjustment for mediator (‘direct effect’)	Percent mediated (‘indirect effect’)
BMI *z*‐score at 2 years		Mean difference (95% confidence interval [CI])	Mean difference (95% CI)	
Maternal depression at 1 year	602	−0.14 (−0.29, 0.00)	−0.13 (−0.28, 0.01)	5.1
Family interaction at 1 year	603	−0.14 (−0.28, 0.00)	−0.13 (−0.28, 0.01)	5.5
Positive discipline strategies at 1.5 years	574	−0.14 (−0.29, 0.01)	−0.14 (−0.29, 0.01)	1.4
Obesity at 2 years		OR (95% CI)	OR (95% CI)	
Maternal depression at 1 year	602	0.52 (0.33, 0.81)	0.53 (0.34, 0.83)	2.2
Family interaction at 1 year	603	0.52 (0.33, 0.81)	0.53 (0.34, 0.83)	2.7
Positive discipline strategies at 1.5 years	574	0.55 (0.35, 0.86)	0.56 (0.35, 0.87)	1.6

## 4 DISCUSSION

We investigated potential moderators and mediators of the effect of the successful POI sleep intervention on BMI *z*‐score and obesity at 2 years of age. All analyses were of an exploratory nature, and thus findings should be interpreted with caution, and with a view to generating hypotheses and informing the development of new research investigating sleep interventions and their effects on childhood obesity outcomes. Some potential moderators were identified: deprivation and ethnicity, where it appeared that the sleep intervention may have been more effective in families living in areas of high deprivation, and in children with mothers of non‐European, non‐Māori ethnicity.

Before considering the findings of the moderation and mediation analyses more closely, it is relevant to note that the overall outcomes reported in Table 2 (lower mean difference BMI *z*‐score and reduced risk of obesity at age 2 years among those who received the sleep intervention compared to those who did not) are important for several reasons. First, the POI sleep intervention was brief, consisting in most cases of only two perinatal parent contacts (group education session in late pregnancy and home visit at 3 weeks post‐partum). While it has been suggested that interventions of long duration may be necessary to create sustained change,^28^ these findings indicate that there is potential for sleep interventions to be brief, economical,^29^ and effective.^12^, ^14^ Second, while the mean difference in BMI *z*‐score at age 2 years may be considered small at −0.15 (95% CI −0.28 to −0.01), it agrees with a recent review of RCTs for the prevention of obesity in infancy, which found that differences in BMI *z*‐scores reported ranged from −0.01 to −0.30.^28^ It has been suggested that in early childhood obesity interventions, differences in mean BMI *z*‐scores of as little as −0.12, if replicated widely, are enough to make important differences at a public health level.^5^ This points to the success of the POI sleep intervention, and to a need to understand why, and for whom, the sleep intervention had a beneficial effect on obesity outcomes. Moderation and mediation analyses are a cost‐effective way of obtaining new insights into potential intervention mechanisms, and are increasingly encouraged in health literature.^30^, ^31^

Very little work appears to have investigated the moderators and mediators of obesity outcomes in early childhood obesity interventions. One Australian exploration of potential moderators and mediators of an online healthy lifestyle programme (incorporating nutrition, physical activity, screen time and sleep for 2–5 year old children) on BMI change did not find any significant moderating or mediating effects.^32^ However, in that study there were also no significant between‐group differences in child BMI at trial completion.^33^ The authors recommended that other childhood obesity interventions also investigate a wide range of factors to allow comparisons between studies and develop a better understanding of the factors contributing to successful interventions.^33^

With regard to potential moderators, several of those identified in the current work, particularly deprivation level and maternal ethnicity, warrant further investigation. In New Zealand, children of Māori ethnicity, and children from more disadvantaged backgrounds, are over‐represented in obesity statistics.^34^ That the POI sleep intervention appeared to be more effective among those living in more deprived areas at 2 years, is potentially promising. However, conversely, while we are reluctant to place emphasis on the ethnicity findings due to the comparatively low numbers in the Māori and ‘Other’ groups, it is possible that the POI sleep intervention was less effective in children of Māori mothers, than in children of mothers with other ethnicities. Notably, the messages in the POI sleep intervention were consistent with existing sleep health recommendations for New Zealand children. In the time period since the POI sleep intervention was completed (8–9 years ago), recognition that some of these recommendations are inconsistent with the worldviews, socio‐cultural contexts and realities of some groups, including Māori whānau (families), has received more attention.^35^, ^36^ We suggest that future sleep interventions aim to recruit a greater range of ethnically and socioeconomically diverse participants, and carefully consider how intervention messages are framed, to ensure that they do not increase inequities in vulnerable populations.^37^

A key strength of this work is that it contributes to a very small, but important and growing area of research. Our findings are intriguing because while some differences in children's sleep behaviours were observed at 1 year of age between children who received the sleep intervention compared to children who did not, these differences were small (overnight sleep efficiency improved by 1.2%, time awake after sleep onset decreased by 8 min and number of night waking decreased by 0.14) and were not maintained at 2 years of age; yet, the sleep intervention clearly improved obesity outcomes at 2 years. Thus, there is a clear need to identify the mechanisms responsible for the effect of the sleep intervention on obesity outcomes to ensure that future research can be targeted to act on them. Sleep interventions also have potential as obesity prevention interventions because sleep may be potentially a less stigmatizing behaviour to focus on than diet and activity.^35^

A key limitation of this work is that all presented analyses are of an exploratory nature, and thus the findings should be interpreted with caution. The lack of identified mediators indicates a need to think more broadly to identify other potential mediators. Weight‐related behaviours such as dietary intake and physical activity did not differ following the intervention^13^, ^16^ . However, the POI study did not measure some parental feeding behaviours that may have contributed to the reductions in obesity observed. For example, the presence or absence of night‐time milk feedings after 6 months of age was not measured. It is possible that the sleep intervention's focus on improving children's self‐regulation of sleep behaviours resulted in less night‐time milk feeding and lower overall energy intake among children who received the sleep intervention compared to children who did not. Thus, future work could focus more on specific parental feeding practices and eating behaviours, which might be influenced by a sleep intervention. Alternatively, it may be that the tools used to measure potential mediating effects in the POI study were not sensitive enough to detect the relatively small changes in energy‐related behaviours that might be required to explain the differences in BMI *z*‐score observed over the course of a 2 year intervention.^38^ Other, more precise measurement techniques may be required. Furthermore, there was considerable homogeneity within the POI study population (mostly New Zealand European families living in areas of low or moderate deprivation), which was particularly evident in the moderation analyses, where numbers for some demographic characteristics were very low.

In conclusion, the exploratory analyses in this paper identified few potential moderators, and no mediators, of the effect of the POI sleep intervention on BMI *z*‐score and obesity at 2 years of age. Deprivation and maternal ethnicity were identified as potential moderators, and should be further considered alongside other potential moderators and mediators in future sleep intervention research in more ethnically and socioeconomically diverse populations.

## CONFLICTS OF INTEREST

No conflict of interest was declared.

## AUTHOR CONTRIBUTIONS

Rachael Taylor and Barry Taylor served as Principal Investigators of the POI study. Rachael Taylor, Barry Taylor, Barbara Galland, Anne‐Louise Heath, Dione Healey, Rachel Sayers, Burt Hatch and Kim Meredith‐Jones designed the project or undertook the research. Rachael Taylor, Jillian Haszard and Louise Fangupo led this manuscript. Jillian Haszard designed and undertook the statistical analysis. Louise Fangupo wrote the first and subsequent drafts of the manuscript. Rachael Taylor had primary responsibility for final content. All authors critically revised the manuscript drafts and read and approved the final manuscript.

## Supporting information


**Figure S1.** The hypothesized pathway in which the obesity outcomes (c) of the POI sleep intervention (a) may be influenced by the potential moderating factors of demographics and study design (b).
**Figure S2**. Potential mediation pathway for “child” and “parent‐household” factors hypothesized to mediate the effect of the sleep intervention on obesity outcomes at 2 years of age, where A=the effect of the sleep intervention on child and parent/household factors at time before the obesity measure, B=the association between potential mediators and obesity outcomes at 2 years of age, and C=the direct effect of the POI Sleep intervention on obesity outcomes at 2 years of age.
