# Supplementary material for: Investigating the moderators and mediators of an effective sleep intervention in the Prevention of Overweight in Infancy (POI) randomized controlled trial: Exploratory analyses
Source: Clin Obes. 2022 Mar 16;12(3):e12516. doi: 10.1111/cob.12516 (PMC9286685; doi:10.1111/cob.12516)
Supplement: Supplementary file 1 — Figure S1. The hypothesized pathway in which the obesity outcomes (c) of the POI sleep intervention (a) may be influenced by the potential moderating factors of demographics and study design (b). Figure S2. Potential mediation pathway for “child” and “parent‐household” factors hypothesized to mediate the effect of the sleep intervention on obesity outcomes at 2 years of age, where A=the effect of the sleep intervention on child and parent/household factors at time before the obesity measure, B=the association between potential mediators and obesity outcomes at 2 years of age, and C=the direct effect of the POI Sleep intervention on obesity outcomes at 2 years of age. [file COB-12-0-s001.docx]

**b. Moderators:**

**Demographic and study design factors**

**c.**

**Obesity outcomes at 2 years of age**

**a.**

**POI Sleep intervention**

**Supporting Figure 1.** The hypothesized pathway in which the obesity outcomes (c) of the POI sleep intervention (a) may be influenced by the potential moderating factors of demographics and study design (b).

**Mediators:**

**“Child” and “parent/household” factors**

**A.**

**B.**

**Obesity outcomes at**

**2 years of age**

**POI Sleep intervention**

**C.**

**Supporting Figure 2.** Potential mediation pathway for “child” and “parent-household” factors hypothesized to mediate the effect of the sleep intervention on obesity outcomes at 2 years of age, where A=the effect of the sleep intervention on child and parent/household factors at time before the obesity measure, B=the association between potential mediators and obesity outcomes at 2 years of age, and C=the direct effect of the POI Sleep intervention on obesity outcomes at 2 years of age.
